# Usefulness of Red Cell Width Distribution (RDW) in the Assessment of Children with Pulmonary Arterial Hypertension (PAH)

**DOI:** 10.1007/s00246-019-02077-4

**Published:** 2019-03-04

**Authors:** Malgorzata Zuk, Anna Migdal, Joanna Dominczak, Grazyna Brzezinska-Rajszys

**Affiliations:** Department of Cardiology, The Children’s Memorial Health Institute, Al. Dzieci Polskich 20, 04-730 Warsaw, Poland

**Keywords:** Pulmonary arterial hypertension, Children, Red cell width distribution, Prognostic marker

## Abstract

Red cell width distribution (RDW) is known to be a prognostic marker in adults with pulmonary hypertension. The value of this test in the pulmonary arterial hypertension (PAH) pediatric population was not yet established. The aim of the study was to evaluate the prognostic value of RDW in children with PAH and utility of this parameter in the management. Data were collected retrospectively in 61 patients with PAH confirmed by right heart catheterization. RDW was measured at diagnosis, 3 and 12 months after initial therapy, during and after deterioration if occurred. Results were compared with NTproBNP, WHO-FC and oxygen blood saturation. Mean RDW at baseline was 15.3 ± 2.4% (12.1–24.4, median 14.7%) and was elevated in 29 patients (47%). There were no significant difference in clinical status, NTproBNP and hemodynamic parameters among patient with normal and elevated RDW at diagnosis. Poor negative correlation with SaO_2_ and SvO_2_ was shown. After 3 and 12 months of treatment no significant change of RDW level was found despite of statistically significant improvement of WHO-FC and decrease of NTproBNP level (NS). Episodes of clinical deterioration weren’t connected with change of RDW level (16 vs. 15.6% NS). Kaplan–Meier analysis did not show differences in prognosis between patients with normal and elevated RDW. Elevation of RDW was not associated with any measured parameters. Prognostic value of RDW in the pediatric PAH population was not confirmed. Usefulness of RDW in management in PAH pediatric population is limited and required further studies.

## Introduction

RDW is one of the parameters in complete blood count (CBC) that measures variation in red blood cell size or volume (anisocytosis). The most common cause of an elevated RDW is anemia [evaluated in association with mean corpuscular volume (MCV) in differential diagnosis]. Regardless of that higher than normal RDW has also been described as a risk factor for unfavourable clinical course in various diseases in children [1–3]. RDW is known to be a prognostic marker in adult with pulmonary hypertension [4–6]. The value of this test in the PAH pediatric population was not yet established. The aim of the study was to evaluate the prognostic value of RDW in children with PAH and utility of this parameter in management.

## Materials and Methods

Results of lab tests performed between 1995 and 2017 in 61 patients with PAH confirmed by right heart catheterization were analyzed retrospectively. Blood count (hematocrit hemoglobin, MCV and RDW) were analyzed at diagnosis (basic results), 3 and 12 months after initial therapy and during clinical worsening and 3 and 12 months later. Patients with anemia (abnormal MCV and Hb below normal range) or infectious disease (anamnesis, elevated biomarkers—leucocytes and/or CRP) were excluded. Clinical worsening (CW) was defined as death or lung transplantation or clinical deterioration requiring treatment intensification (new medication, Potts shunt, atrioseptostomy)—the first event was used to calculate the time to clinical worsening. RDW was compared with NTproBNP, WHO-FC and oxygen blood saturation (SaO_2_). WHO-FC was analyzed in two groups I/II WHO-FC (low risk) and III/IV WHO-FC (high risk).

Additionally because of possible influence of cyanosis and hyperviscosity on RDW data of patients with Eisenmenger syndrome (defined as shunt and rest blood saturation below 90% HbO_2_) including blood saturation, hematocrit, hemoglobin, MCV and RDW were compared with data of other patients.

According to basic results patients were divided into two groups: with normal (N) and elevated (E) RDW. Normal value for RDW in children below 12 years depends on age, for the study it was adopted according to Novak [7] (Table 1). Upper limit was established as mean + 2SD. For children above 12 years normal range for adults 11–14.5% was used.

**Table 1:** Age-appropriate values for RDW (Novak [7])

Age	RDW (mean ± SD)
1–6 months	13.0 ± 1.5
7–12 months	13.7 ± 0.9
13–24 months	13.4 ± 1.0
2–3 year	13.2 ± 0.8
4–5 year	12.7 ± 0.9
6–8 year	12.6 ± 0.8
9–11 year	12.8 ± 1.0

Comparison between two groups included: age, gender, diagnosis, presence of Down syndrome, shunt (from significant, contributing to PAH development, to even small like PFO), Eisenmenger syndrome and data from right heart catheterization. Hemodynamic parameters for analysis (SvO_2_, mRAP, PVRI, CI, mPAP/mSAP) were chosen according to previous studies in children because of influence on prognosis [8].

Impact of initial RDW on prognosis and relation between RDW and clinical course (worsening or improvement after treatment) were evaluated. The initial results did not confirm difference in RDW between Eisenmenger and no Eisenmenger group in study population. Due to too small number of patients, they were not analyzed separately.

Cumulative incidence and survival probability were estimated in both group and compared. Impact of RDW on risk of clinical worsening and death was evaluated. The same survival analyses were performed after excluding children with Eisenmenger syndrome, because of known better prognosis in these patients. The results of survival analyses depending on RDW were compared with results of survival analyses in patients with normal and elevated NTproBNP, which is confirmed prognostic factor in children with PH. Based on previous studies [9] value 605 pg/ml was taken as a cut-off level.

Data were collected retrospectively and contained results of examinations routinely performed in patients with pulmonary hypertension. For all procedures informed consent from parents and older children was obtained.

### Statistical Analysis

Descriptive data were presented as mean ± standard deviation, and median or percentage if necessary. Normal distribution was determined using Shapiro–Wilk test. To compare distribution in groups parametric (Student’s *t* test for independent or dependent samples) and non parametric (Whitney–Mann and Friedman’s) tests were used. To assess a differences between groups in the case of qualitative variables Chi square test of Pearson with Yates corrections where necessary was performed. Association between two quantities was estimated using the Spearman's correlation coefficient (rho).

The survival analysis was performed using Kaplan–Meier survival function estimator. The results were shown in the survival curves. To evaluate the significance of the difference between two survival functions a log-rank test was used. Cumulative incidence analysis of clinical worsening was done, to compare probability in two groups Gray test was used. The results were shown in the cumulative incidence curves. To assess impact for risk, single Cox proportional hazards model was used. In all analyses, the level of significance *p* = 0.05 was adopted. The analyses were done in the XLSTAT software.

## Results

Study group counted 61 patients with PAH, 16 of them have Eisenmenger syndrome. Mean RDW at baseline was 15.3 ± 2.4% (12.1–24.4, median 14.7%), after exclusion of Eisenmenger patients 15.1 ± 2.5% (13.9–18, median 14.4%). There was no statistically significant difference in RDW value between both group. Additionally, difference in RDW value between patient with Eisenmenger syndrome (*N* = 16) and rest of group (*N* = 45) wasn’t observed (15.7 ± 2.5 vs. 15.1 ± 2.5, NS) despite significantly lower blood saturation (83 ± 4 vs. 95 ± 3%HbO_2_, *p* < 0.01) and higher hemoglobin (16.1 ± 0.8 vs. 14.2 ± 0.2%HbO_2_, *p* = 0.03) and hematocrit (48.8 ± 2.2 vs. 42.8 ± 0.8%, *p* = 0.01) in Eisenmenger patients. MCV was similar in both group (89.8 ± 2.3 vs. 87.0 ± 0.9 fl, NS).

Demographic, clinical and hemodynamic data at diagnosis are shown in Table 2. No correlation between RDW and NTproBNP or hemodynamic parameters was observed. Poor negative correlation with SaO_2_ (*ρ* − 0.26, *p* = 0.05) and SvO_2_ (*ρ* − 0.36, *p* = 0.01) was shown. There was no difference in RDW between patients with WHO-FC I/II and WHO-FC III/IV (15 ± 2% vs. 15.5 ± 3%, NS).

**Table 2:** Demographic, clinical and hemodynamic data at diagnosis

Parameter	Unit	All	Normal RDW	Elevated RDW
N/RDW (%)		61 15.3 ± 2.4; 14.7	32 13.7 ± 0.8; 13.8	29 17.0 ± 2.5; 16.0
Age	Years	7.5 ± 6.0 5.9	8.4 ± 6.3 7.6	6.5 ± 5.9 3.9
Gender	Male/female	24/37	11/21	13/16
Clinical worsening	N (counted only 1st event)	31	15	16
	Death/lung transplantation/deterioration	18/3/17	8/2/7	10/1/10
	1 year without CW	31	20	11
Down syndrome		20	9	11
Diagnosis	IPAH + FPAH	25	14	11
	APAH–CHD	20	9	11
	APAH–CHD after surgery	16	9	7
Shunt		38	17	21
Eisenmenger syndrome (shunt + SaO_2_ < 90%HbO_2_)		16	6	10
WHO FC	I/II/III/IV	0/34/24/3	0/17/12/3	0/17/12/0
SaO_2_	%HbO_2_	92 ± 6 95	91 ± 9 95	88 ± 10 93
NTproBNP	pg/ml	1769 ± 2110 889	1488 ± 1782 572	2161 ± 2497 1056
Hb	g/dl	14.7 ± 2.3 14.5	14.7 ± 1.9 14.5	14.9 ± 2.7 14.2
MCV	fl	87.6 ± 7.0 87.6	88.4 ± 6.1 88.7	86.8 ± 7.9 85.4
SvO_2_	%HbO_2_	66 ± 9 67	67 ± 11 69	64 ± 5 66
mRAP	mmHg	9 ± 4 9	9 ± 4 9	10 ± 4 10
mPAP	mmHg	59 ± 14 57	57 ± 15 61	60 ± 13 57
mPAP/mSAP		0.94 ± 0.25; 0.93	0.91 ± 0.24; 0.89	0.97 ± 0.23; 0.95
CI	l/min/m^2^	3.4 ± 1.5 3.1	3.3 ± 1.1 3.1	3.6 ± 2.0 2.9
PVRI	WU m^2^	16.1 ± 9.5 13.5	16.2 ± 11.8 12.1	14.9 ± 6.5 13.3

*CW* clinical worsening, *IPAH* idiopathic pulmonary arterial hypertension, *FPAH* familial pulmonary arterial hypertension, *APAH–CHD* pulmonary arterial hypertension associated with congenital heart defect, *SaO2* blood oxygen saturation, *WHO-FC WHO* functional class, *NTproBNP* N-terminal pro-brain natriuretic peptide, *HB* haemoglobin, *MCV* mean corpuscular volume, *SvO2* mixed venous oxygen saturation, *mRAP* mean right atrial pressure, *mPAP* mean pulmonary arterial pressure, *mSAP* mean systemic arterial pressure, *CI* cardiac index, *PVRI* pulmonary vascular resistance index

Normal RDW in basic evaluation was found in 32 patients (N group, mean RDW 13.7 ± 0.8%), elevated in 29 (E group, mean RDW 17.0 ± 2.5). The comparison between groups was shown in Table 2. There were no statistically significant differences between groups in studied parameters.

Data of 31 patients without CW at least 12-month follow-up were analyzed. There were ten patients with initially elevated RDW (E) and 21 with normal (N). In whole group after 3 and 12 months of treatment, no change of RDW level was found despite of statistically significant improvement in WHO-FC and tend to decrease of NTproBNP level (NS) (Table 3).

**Table 3:** Comparison of data at diagnosis and after 3 and 12-month follow-up

Parameter	Units	0	3 months	12 months	*p*
RDW	%	14.8 ± 1.8 14.7	15.2 ± 2.5 14.9	15.4 ± 3.3 14.1	NS
SaO_2_	%HbO_2_	91 ± 7 94	91 ± 7 93	91 ± 8 95	NS
WHO-FC	I, II vs. III, IV	**17**/**14**	**26**/**5**	**26**/**5**	**0.01**
NTproBNP	pg/ml	1893 ± 2504 528	1147 ± 2125 355	773 ± 1201 228	NS

Bold values—statistically significant differences

In both initial RDW-dependent groups (N and E) expected treatment response was observed. Number of patients with high-risk WHO-FC (III/IV) diminished after 1-year follow-up from 4 to 2 in group E (40–20%) and from 10 to 2 in group N (47–9%). NTproBNP changed in group E from 2596 ± 3233 pg/ml to 392 ± 282 pg/ml and in group N from 1212 ± 1645 pg/ml to 704 ± 1030 pg/ml. Because of too small groups statistical analysis was not performed.

In 31 patients, clinical worsening was observed in any time of follow-up: deterioration requiring treatment intensification in 17 and/or death in 18 and/or lung transplantation in 3. 15 of them had normal RDW at diagnosis (basic RDW), and 16 elevated (NS). There was significant difference between patients with no CW and with CW during follow-up in NTpro BNP level (1169 ± 2020 pg/ml vs. 2399 ± 2062 pg/ml; *p* < 0.01), but difference in value of basic RDW (14.9 ± 2.1% vs. 15.7 ± 2.7%; NS) wasn’t observed.

**Fig. 1:**
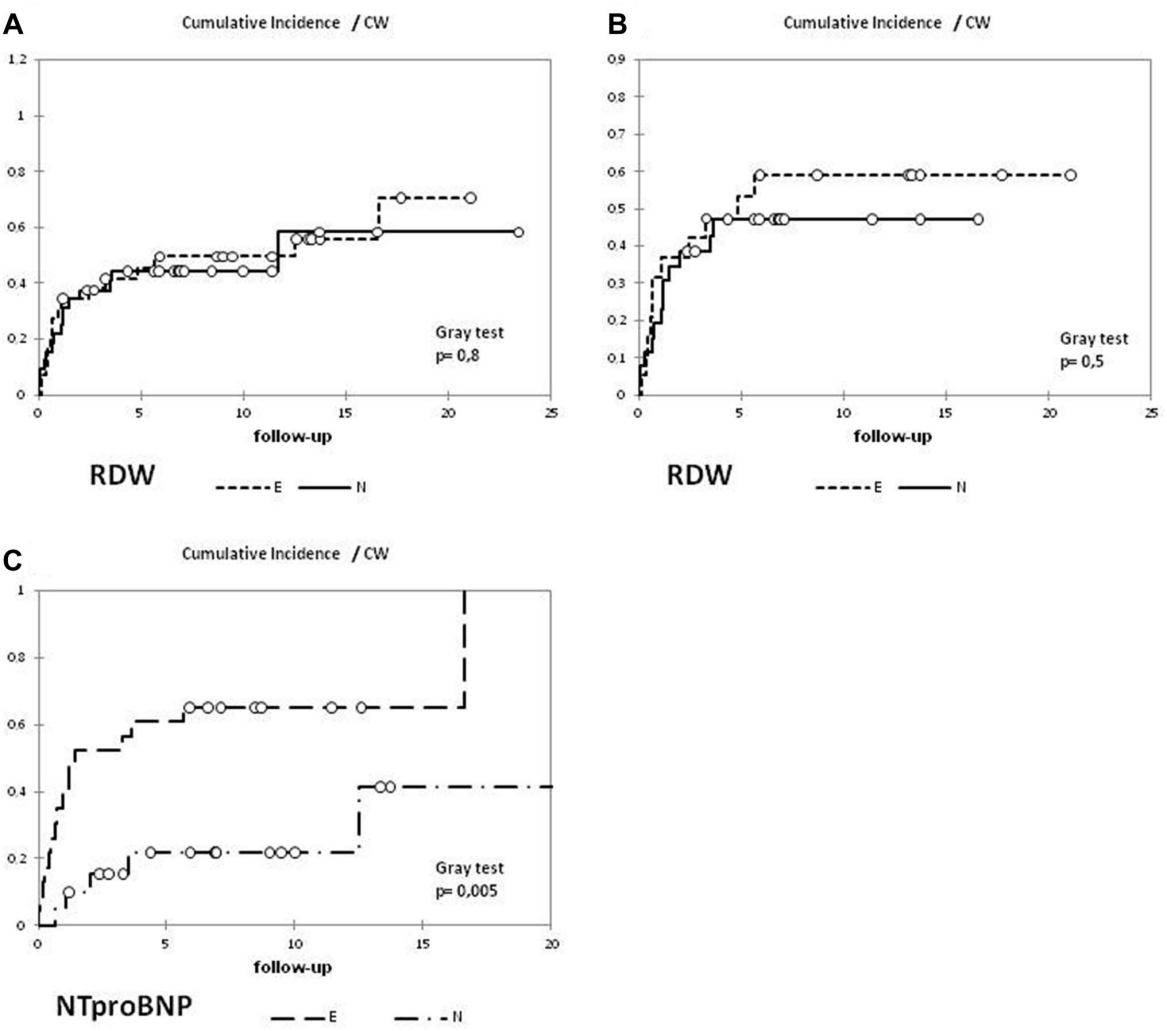
Cumulative incidence of events comprising patients with initially normal and elevated RDW (**a** all patients, **b** after exclusion of patients with Eisenmenger syndrome). For comparison, cumulative incidence depending on NTproBNP (**c**) in the same group. (E-elevated, N-normal value)

1–3–5–15 years cumulative incidence of CW was, respectively, 28.0–38.0–45.0–56%. The incidence of CW was compared in patients with initially normal and elevated RDW (Fig. 1)—there was no significant difference in whole material and after excluding Eisenmenger patients. In univariate analysis, influence of RDW at diagnosis on risk of clinical worsening was not confirmed at whole studied population (HR 1.079; 95% CI 0.94–1.23) and after exclusion of patients with Eisenmenger syndrome (HR 1.088; 95% CI 0.94–1.26).

Clinical deterioration requiring treatment intensification occurred in 17 patients. It was not connected with significant RDW changes (16.0 ± 2.0% vs 15.6 ± 2.5%, NS) although significant worsening in WHO-FC and NTpro BNP was observed (Table 4).

**Table 4:** Comparison of data at diagnosis and during deterioration

Parameter	Units	0	Deterioration	*p*
RDW	%	16.0 ± 2.0 16.0	15.6 ± 2.5 15.1	NS
SaO_2_	%HbO_2_	93 ± 6 95	90 ± 7 88	NS
WHO-FC	I, II vs. III, IV	**11**/**6**	**3**/**14**	< **0.01**
NTproBNP	pg/ml	**2446** ± **2860** **1384**	**4438** ± **4690** **2717**	**0.01**

Bold values—statistically significant differences

**Fig. 2:**
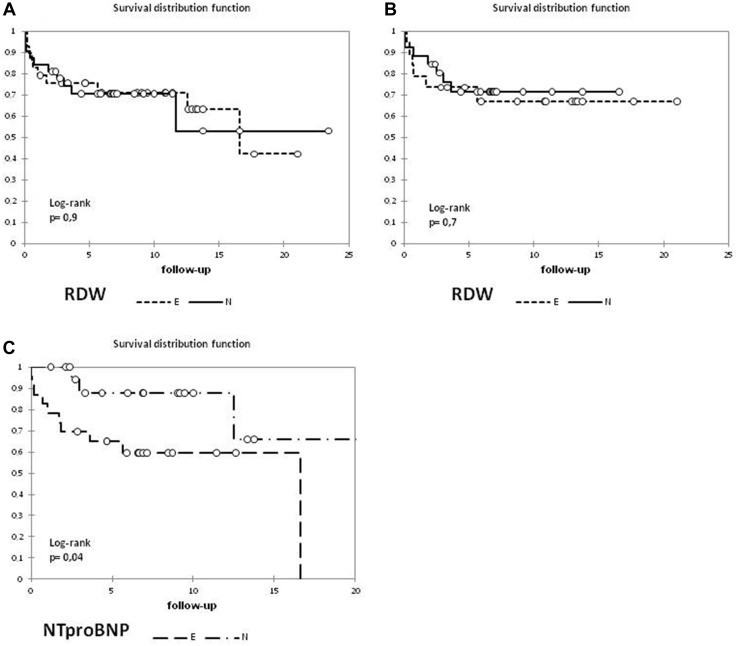
Kaplan–Meyer estimation of survival comprising patients with initially normal and elevated RDW (**a** all patients, **b** after exclusion of patients with Eisenmenger syndrome). For comparison survival curves depending on NTproBNP (**c**) in the same group. (E-elevated, N-normal value)

Twenty patients died or at least required lung transplantation. In the entire group, 1-3-5-15-years survival rate was, respectively, 82-75-73-60%. There was no difference between estimated survival in patients with initially normal and elevated RDW in whole material and after excluding Eisenmenger patients (Fig. 2). In univariate analysis, influence of RDW at diagnosis on risk of death was not confirmed in whole studied population (HR 1.020; 95% CI 0.86–1.21) and after exclusion of patients with Eisenmenger syndrome (HR 1.082; 95% CI 0.88–1.33).

## Discussion

Management of pulmonary hypertension in children is derived from adult pulmonary hypertension studies. Prognostic value of PAH biomarkers confirmed in adult population should be verified in children. It regards especially nonspecific biomarkers like RDW [10]. However, there are some data concern RDW as a risk factor in various pathologies in children, but in pediatric pulmonary hypertension such researches were not performed. According to our knowledge, this is the first study that evaluated this biomarker in children with PAH.

RDW is a known prognostic biomarker in adults with various pulmonary hypertension forms. Hampole et al. confirmed that RDW was independent predictor of mortality in population of 162 adults with PAH [4]. Rhodes et al. studied potential circulating prognostic biomarkers in IPAH patients. They found that RDW was related to disease severity. Addition of RDW to measurement of level NTproBNP and exercise capacity significantly increased their prognostic value [5]. In our study, although a significantly higher NT-BNP level was observed in patients with clinical worsening during follow-up, RDW remained unchanged regardless of the clinical course. In contrast to results cited above, RDW was not confirmed as a prognostic marker in our study population.

Smukowska-Gorynia et al. suggested that RDW was a biomarker of good treatment response and better prognosis [6]. In our material, the initiation of treatment was associated with clinical improvement and a decrease of NTproBNP level, but not with a change in RDW value.

Increased RDW results from ineffective erythropoesis (iron-deficiency anaemia) or hemolysis (inflammation, oxidative stress) [2]. Those clinical states are frequent in paediatric population, what could influence on RDW results in children with PH. To minimalize this bias in our study patients during infection and with anaemia were excluded. Normal range of MCV in whole group suggests no iron deficiency. It appears that known iron influence on regulation of pulmonary vascular tone [11] was eliminated.

Higher erythropoesis due to hypoxia can also lead to increased RDW [12]. In our material, expected negative correlation of RDW and blood saturation was observed. In the group of patients with Eisenmenger syndrome, despite significantly lower mean blood saturation and higher mean hemoglobin and hematocrit RDW was similar to other patients. Yang et al. found correlation between RDW and desaturation and SvO_2_ in Eisenmenger patients [13]. Additionally, they stated that RDW was an independent prognostic marker and was correlated with hemodynamic parameters (mPAP, PVRI). Those conclusions were not confirmed in our material but it included only 26% patients with Eisenmenger syndrome. Because of different (better) prognosis in these patients, all survival analysis were performed for whole group and after exclusion children with Eisenmenger syndrome. It did not affect on results.

Difference between our results and results published in adult population were considered. Two possible causes were mentioned. First, in other studies, the most common causes of RDW elevation such as anaemia and inflammation were not excluded. It is possible that these additional diseases not RDW value had influence on clinic course. Second reason is that RDW is a nonspecific marker and many factors can affect its value especially in children. Because of the retrospective character of study, less common reasons of RDW elevation could not be eliminated. Further studies are necessary.

## Conclusions

In study group association of RDW value with hemodynamic parameters, Eisenmenger syndrome, NTproBNP level, WHO-FC and change of clinical status (improvement or deterioration) was not shown. Prognostic value of RDW in the paediatric PAH population was not confirmed. Usefulness of RDW in management in PAH paediatric population is limited.

## Abbreviations

CBC: Complete blood count
CI: Cardiac Index
CW: Clinical worsening
HB: Hemoglobin
MCV: Mean corpuscular volume
mPAP: Mean pulmonary arterial pressure
mRAP: Mean right atrial pressure
mSAP: Mean systemic arterial pressure
NTproBNP: N-terminal pro-brain natriuretic peptide
PAH: Pulmonary arterial hypertension
PVRI: Pulmonary vascular resistance index
RDW: Red cell width distribution
SaO_2_: Blood oxygen saturation
SvO_2_: Mixed venous oxygen saturation
WHO-FC: WHO functional class
